# Optimal Design of Intervention Studies to Prevent Influenza in Healthy Cohorts

**DOI:** 10.1371/journal.pone.0035166

**Published:** 2012-04-13

**Authors:** Brendan Klick, Hiroshi Nishiura, Benjamin J. Cowling

**Affiliations:** 1 Infectious Disease Epidemiology Group, School of Public Health, Li Ka Shing Faculty of Medicine, The University of Hong Kong, Pokfulam, Hong Kong Special Administrative Region, People's Republic of China; 2 PRESTO, Japan Science and Technology Agency, Saitama, Japan; Public Health Agency of Canada, Canada

## Abstract

**Background:**

Influenza cohort studies, in which participants are monitored for infection over an epidemic period, are invaluable in assessing the effectiveness of control measures such as vaccination, antiviral prophylaxis and non-pharmaceutical interventions (NPIs). Influenza infections and illnesses can be identified through a number of approaches with different costs and logistical requirements.

**Methodology and Principal Findings:**

In the context of a randomized controlled trial of an NPI with a constrained budget, we used a simulation approach to examine which approaches to measuring outcomes could provide greater statistical power to identify an effective intervention against confirmed influenza. We found that for a short epidemic season, the optimal design was to collect respiratory specimens at biweekly intervals, as well as following report of acute respiratory illness (ARI), for virologic testing by reverse transcription polymerase chain reaction (RT-PCR). Collection of respiratory specimens only from individuals reporting ARI was also an efficient design particularly for studies in settings with longer periods of influenza activity. Collection of specimens only from individuals reporting a febrile ARI was less efficient. Collection and testing of sera before and after influenza activity appeared to be inferior to collection of respiratory specimens for RT-PCR confirmation of acute infections. The performance of RT-PCR was robust to uncertainty in the costs and diagnostic performance of RT-PCR and serological tests.

**Conclusions and Significance:**

Our results suggest that unless the sensitivity or specificity of serology can be increased RT-PCR will remain as the preferable outcome measure in NPI studies. Routine collection of specimens for RT-PCR testing even when study participants do not report acute respiratory illness appears to be the most cost efficient design under most scenarios.

## Introduction

Influenza is a major cause of mortality and morbidity worldwide [1]. Field studies of influenza have been invaluable for understanding influenza epidemiology [2], [3]. In a ‘healthy cohort’ study, participants are enrolled in a defined period, usually before the start of an influenza epidemic season, and are followed up to measure the incidence of influenza infections and illnesses usually through one or more entire epidemics. Many randomized controlled trials (RCTs) studying the efficacy of influenza vaccinations [4], [5], [6], [7], [8], [9], [10], [11], antiviral prophylaxis [12], [13], [14], [15], [16], [17], [18], [19], and non-pharmaceutical interventions [20], [21], [22], [23], [24], [25] at preventing influenza infection and illness have followed healthy cohort study designs.

There are a variety of ways to identify influenza infections in cohorts (Table 1) [26], [27], [28], [29]. Because acute upper respiratory tract infections (URTIs) associated with different pathogens can have similar clinical presentation, the sensitivity and specificity of syndromic classifications of influenza infection, or proxy measures such as absenteeism, tend to be poor when compared to laboratory-based outcomes [26]. Respiratory specimens, such as nasal swabs or aspirates, can be collected from patients with acute URTI for virologic testing. These methods have high specificity and high sensitivity to correctly identify acute influenza virus infections and exclude other causes, with reverse transcription polymerase chain reaction (RT-PCR) recently superseding viral culture as the gold standard virologic approach [29], [30]. However, due to the relatively short duration of influenza viral shedding during acute course of infection, respiratory specimens should be collected within 3–5 days of illness onset [31], [32], [33], although asymptomatic infections can also be identified by virologic testing [31], [32]. Because collection and testing of respiratory specimens is costly, most cohort studies using virologic outcomes have collected respiratory specimens only following onset of illness in subjects [5], [6], [23] although it could be feasible to arrange routine collection of specimens at regular intervals. Consequently, if RT-PCR is to be used as the main influenza diagnostic method in cohort studies, considerable resources must be devoted to the timely identification of illnesses in subjects and collection of respiratory specimens. In contrast, serologic testing of paired sera collected before and after epidemic periods of influenza activity can be easier to coordinate, and rises in antibody titers during the influenza season can indicate recent infection albeit with lower sensitivity and specificity than RT-PCR [26] and with specific limitations for identifying infections in recipients of influenza vaccine because of falling antibody titers after vaccination [30], [34].

**Table 1:** Approaches to identify influenza infection and illness or their correlates in community-based studies.

Category	Approach	Advantages	Disadvantages
Serologic confirmation	Paired sera taken before and after the influenza season with four-fold rise in antibody titers typically used as evidence of infection.	• Generally high sensitivity and specificity to identify infections	• Collection of sera is invasive• Requires laboratory expertise• Not all infections are associated with rises in antibody titers (i.e. imperfect sensitivity)• Cross-reactive antibody responses can be associated with a lack of specificity
Virologic confirmation	RT-PCR analysis of throat or nose swabs	• Gold standard for diagnosis of influenza infection	• Requires a respiratory specimen collected within 3–5 days of symptom onset [32], [33]
	Viral culture	• Virus is recoverable for further analysis [48]	• Expensive• Time intensive [48]
	Rapid antigen test	• Fast—gives results within hours	• Lower sensitivity than viral culture or RT-PCR [49], [50], [51]
Clinical outcomes	Hospitalisations associated with confirmed influenza	• Confirmed infection of clinical importance	• Rare event so is a low-powered endpoint
	Hospitalisations associated with influenza-like illness	• Outcome of clinical importance	• Rare event so is a low-powered endpoint
	Outpatient consultations associated with confirmed influenza	• Confirmed infection of clinical importance	• Misses less serious influenza infections [20]
Proxy outcomes	Absenteeism	Easy to collect data especially in school or workplace [52]	Not influenza specific
Based on reported signs and symptoms	Acute respiratory illness (ARI), an acute upper respiratory tract infection which is not necessarily associated with febrile illness; one common definition is at least two of body temperature ≥37.8°C, cough, headache, sore throat, phlegm or myalgia [26], [27]	• Does not require clinical specimens or laboratory tests• Higher sensitivity than FARI	• Lower specificity than FARI• Lower sensitivity and specificity than laboratory confirmed outcomes
	A febrile ARI (FARI), an acute upper respiratory tract infection, one common definition is body temperature ≥37.8°C plus cough or sore throat [26], [27]	• Does not require clinical specimens or laboratory tests• Higher specificity than ARI	• Lower sensitivity and specificity than laboratory confirmed outcomes

While timely collection of respiratory specimens allows sensitive identification of influenza infections, many acute URTIs are not caused by influenza virus infections [35], [36], [37]. Therefore, when planning to collect specimens for RT-PCR testing following report of an acute URTI, it is important to consider the expected incidence rates of URTIs with various presentations in the fieldwork budget. Within a constrained budget, the costs of collection and testing of specimens has to be offset against the number of participants enrolled in the study, but this can be problematic given that the incidence of influenza and other respiratory diseases varies from year to year. If the incidence of influenza and other respiratory diseases is lower than expected and fewer specimens are collected, some funds for testing may remain unspent, while if activity is higher, funding may be insufficient to collect and test specimens from all illnesses.

It is unclear which laboratory approach – serology or RT-PCR – is the most cost effective and efficient for use in intervention studies based on healthy cohort designs. Selecting the best design to maximize study power given fixed available resources is part of Good Clinical Practice and suboptimal designs could waste valuable resources or inconvenience patients unnecessarily. Our objective here is to identify cohort study designs which can maximize statistical power to detect a difference between an intervention and a control arm in preventing influenza infections.

## Methods

We simulated a comparative study for a ‘healthy cohort’ of participants that are not infected with influenza at recruitment. We assume that participants in the cohort are individually randomized in equal proportions between an intervention arm and a control arm (or between two intervention arms), as balanced studies tend to have greater statistical power than unbalanced studies. We proceed under the assumption that the intervention being considered is an NPI, such as wearing face masks or shields, or increasing their hand hygiene behaviors. We assume that all participants are recruited independently and are not members of the same households, schools, or otherwise clustered.

A range of syndromic definitions have been used as proxy outcomes in influenza studies, including definitions aiming at greater sensitivity such as “acute respiratory illness” (ARI) defined as any two of a range of respiratory and systemic symptoms (e.g. fever ≥37.8°C, cough, headache, sore throat, or myalgia) as well as definitions aiming at greater specificity by restricting to febrile ARI (FARI) for example the CDC surveillance definition of “influenza-like illness” as fever ≥37.8°C plus cough or sore throat [20], [26], [27], [38].

We consider seven alternative approaches to identification of influenza infections in the comparative study:

1. Collection and testing by RT-PCR of respiratory specimens from participants reporting FARI.
2. Collection and testing by RT-PCR of respiratory specimens from participants reporting ARI.
3. Collection and testing by RT-PCR of respiratory specimens collected from all participants at biweekly intervals regardless of illness, as well as from any participants reporting ARI.
4. Collection of paired serum from all participants plus collection and testing by RT-PCR of respiratory specimens from participants reporting FARI.
5. Collection of paired serum from all participants plus collection and testing by RT-PCR of respiratory specimens from participants reporting ARI.
6. Collection of paired serum from all participants plus collection and testing by RT-PCR of respiratory specimens collected from all participants at biweekly intervals regardless of illness, as well as from any participants reporting ARI.
7. Collection of paired serum from all participants but no collection of respiratory specimens.

In these approaches, ARI and FARI trigger refers to collection of respiratory specimens within 1–3 days of onset of illness only if and when ARI or FARI are reported by a study participant. Because our interest is in studies that can demonstrate effectiveness of interventions against influenza specifically, we did not consider ARI or FARI as primary outcomes in our analysis and therefore of primary relevance to the present optimal design considerations, although they might be included as secondary outcomes. For analysis of paired sera a 4-fold or greater rise in antibody titers on hemagglutination inhibition (HI) assays is used to indicate infection [26]. We did not consider proxy outcomes such as absenteeism, or clinical outcomes such as hospital admissions or outpatient visits because they were believed to have low power as study endpoints [20], [26].

Table 1 shows the parameter values used in our simulations. We assumed that the intervention could reduce the risk of influenza virus infections by 30%, with a consequent reduction in the rates of ARI and FARI episodes associated with influenza. Our simulations also allowed for an effect of the NPI on the rates of ARI and FARI episodes not associated with influenza [39], [40], [41], [42]. For simplicity we assume that the risk of ARI and FARI associated with non-influenza infections is independent of the transmission dynamics of and infection with influenza virus and vice versa. For each study design variant, we used a Monte Carlo approach to randomly simulate a set of 2500 datasets. For each dataset we used chi-squared tests of the difference between arms in the proportion of laboratory confirmed infections. The proportion of datasets in which the null-hypothesis of no difference was rejected at the 0.05 significance level was defined as the statistical power [33], [43]. Further technical details are provided in Text S1.

For each study budget, we calculated the number of participants per arm that can be recruited given the chosen diagnostic method and consequent costs of follow-up, as well as the anticipated ‘base case’ level of ARI and FARI incidence. We investigated the effect on study power to variability in the activity of influenza and other respiratory viruses during the study as a key sensitivity analysis. This was done because in the case of respiratory specimen collection triggered by ARI or FARI, the number of specimens collected could exceed the allotted budget if the activity of influenza and other non-influenza respiratory viruses was higher than anticipated. If that occurred in our simulation, only the number of specimens allowed by the study budget was tested. Simulations were performed assuming three different scenarios. In the first scenario (I) we assume that the cumulative incidence of ARI and FARI not associated with influenza in the control arm are 0.40 and 0.10 respectively and that these are correctly estimated in advance of the study. In the scenarios (II) and (III) the cumulative incidence of non-influenza ARI and FARI are again 0.40 or 0.10 but for purposes of study planning these are believed incorrectly to be 0.20 and 0.06 or 0.60 and 0.14 respectively. Scenarios II and III are used to illustrate how underestimation or over-estimation of ARI and FARI attack rates will reduce the power of detection methods relying on ARI or FARI report or trigger. Power, sample size, and cumulative incidence of infection in the control arm (i.e. proportion of control arm participants identified as having influenza) were plotted as a function of field budget for these three scenarios.

### Sensitivity Analyses

Due to uncertainties in model parameters, we performed several sensitivity analyses to examine how sensitive power estimates were to variations in model parameters (Table 2). Specifically, we examined the sensitivity of power estimates to differing influenza cumulative incidences, the effect of the NPI intervention on the rate of non-influenza ARI and FARI, the cost of RT-PCR testing, the cost of serological testing, the sensitivity of RT-PCR testing and the sensitivity and specificity of serology. In another sensitivity analysis, we assumed a longer, six-month influenza season with lower incidence rates but the same cumulative incidence of infection across the study as the base case.

**Table 2:** Parameter values and ranges of the input values in sensitivity analysis.

Parameter	Value	Sensitivity analysis	Source
Length of the study	2 months		assumed
Primary cumulative incidence (control)	0.15	0.1, 0.3	assumed
Treatment efficacy	0.30		assumed
Package cost of enrollment of a subject	US$500		(B. J. Cowling, personal communication)
Package cost of collection of a respiratory specimen from a subject and testing by RT-PCR	US$65	US$35, US$130	(B. J. Cowling, personal communication)
Package cost of collection of paired serology from a subject and testing by hemagglutination inhibition	US$130	US$65, US$280	(B. J. Cowling, personal communication)
Serology sensitivity	0.84	0.76, 0.92	[29], [53]
Serology specificity	0.88	0.80, 0.96	[29]
RT-PCR sensitivity	Various depending on timing	Area under the curve decreased by 20% and increased by 10%	[33]
RT-PCR specificity	0.99		[33]
ARI sensitivity for case-ascertained studies	0.68		[33]
FARI sensitivity for case-ascertained studies	0.40		[33]
Reporting rate for cohort studies as compared to case-ascertained studies	0.70		assumed
Control Non-influenza ARI rate	0.40		[6]
Control Non-influenza FARI rate	0.10		[6]
Reduction in rate of non-influenza ARI and FARI	0.15	0, 0.30	assumed

## Results

**Figure 1:**
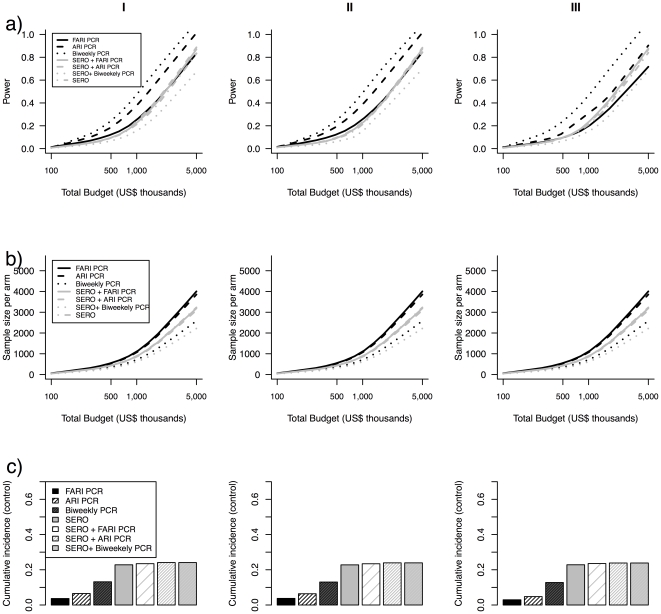
Comparison of alternative study designs. In the plot, the three rows indicate: (A) power, (B) total sample size per arm, and (C) estimated cumulative incidence of influenza in the control arm. Scenario I assumes the unbiased control non-influenza attack ARI and FARI rates are 0.4 and 0.1 respectively which exactly correspond to estimates made in advance of the study. Scenario II assumes the unbiased control non-influenza attack ARI and FARI rates are 0.4 and 0.1 but are underestimated at 0.2 and 0.06 when planning the study. Scenario III assumes the unbiased control non-influenza attack ARI and FARI rates are 0.4 and 0.1 and but are overestimated at 0.6 and 0.14 when planning the study. Control arm cumulative incidence proportion refers to the expected proportion of participants identified as having influenza infection among the control arm. “Combined” refers to paired serology analyzed by HAI plus RT-PCR upon ARI trigger. Black lines are used to denote design variants using RT-PCR confirmation. Grey lines are used to denote design variants using serologic confirmation or serologic plus RT-PCR confirmation.

Model results for the base case parameters are shown in Figure 1 for Scenarios I-III. A summary of the results is given in Table 3. In all scenarios, biweekly RT-PCR plus trigger yielded greater power than other study designs. In Scenario I in which expected rates of ARI and FARI noise matched actual rates, biweekly RT-PCR plus ARI trigger followed by RT-PCR upon ARI trigger were the most powerful study designs. The higher power of biweekly RT-PCR plus ARI trigger was robust to underestimation or overestimation of ARI and FARI rates (Scenarios II and III). Study design variants relying on RT-PCR generally had higher power than serological testing despite having much lower cumulative incidence of confirmed influenza (Figure 1) although in all scenarios RT-PCR upon FARI trigger performed worse or equal in terms of statistical power to serology or the combination of serology and RT-PCR. Cumulative incidence of confirmed influenza in the control arms were highest for study designs that involved serology, and lowest for the study design based on collection of specimens upon FARI trigger.

**Table 3:** Sample size per arm and total budget needed to achieve 80% power for differing methods of identification of influenza infections for both short (2 months) and long (6 months) influenza seasons.

	hort Season		ong season	
Parameter	N	Budget ($)	N	Budget ($)
(#1) Collection and testing by RT-PCR of respiratory specimens from participants reporting FARI.	4218	4,700,000	4218	4,700,000
(#2) Collection and testing by RT-PCR of respiratory specimens from participants reporting ARI.	2610	3,400,000	2610	3,400,000
(#3) Collection and testing by RT-PCR of respiratory specimens collected from all participants at biweekly intervals regardless of illness, as well as from any participants reporting ARI.	1352	2,500,000	1305	3,600,000
(#4) Collection of paired serum from all participants plus collection and testing by RT-PCR of respiratory specimens from participants reporting FARI.	3217	4,000,000	3217	4,000,000
(#5) Collection of paired serum from all participants plus collection and testing by RT-PCR of respiratory specimens from participants reporting ARI.	3027	4,200,000	3027	4,200,000
(#6) Collection of paired serum from all participants plus collection and testing by RT-PCR of respiratory specimens collected from all participants at biweekly intervals regardless of illness, as well as from any participants reporting ARI.	3123	5,700,000	2494	7,500,000
(#7) Collection of paired serum from all participants but no collection of respiratory specimens.	3457	4,800,000	3475	4,800,000

### Sensitivity Analyses

Figure S1, Figure S2, Figure S3, Figure S4, Figure S5, and Figure S6 examine the sensitivity of observed statistical power to cumulative incidence of influenza infection, to the effectiveness of the intervention to reduce non-influenza ARI and FARI, the cost of RT-PCR, the cost of serology, the sensitivity of RT-PCR, and the sensitivity and specificity of serology respectively. Figure S7 shows the effect of increasing the follow-up time from 2 months to 6 months without changing the overall cumulative incidence of infection. In these illustrations, biweekly RT-PCR plus ARI trigger yielded the greatest power except for two cases: serology was similar to biweekly RT-PCR plus ARI trigger when the sensitivity and specificity of serological testing were increased, and RT-PCR upon ARI trigger performed the best for a longer six-month follow-up period. When the cost of serological testing was reduced, the cost of RT-PCR was increased or the sensitivity of RT-PCR was reduced, designs relying on RT-PCR still appeared to outperform serology indicating that RT-PCR is generally more efficient unless the sensitivity and specificity of serology can be increased.

## Discussion

We found that study design variants based on collection of respiratory specimens for RT-PCR testing almost always performed better than study design variants based on serology. Unless the duration of influenza activity was greater than two months, biweekly RT-PCR plus RT-PCR upon ARI trigger was not dominated by any other method. It should be noted that study design variants relying on serological testing had lower statistical power than RT-PCR despite being able to identify a greater proportion of influenza infections. This is consistent with several studies [5], [6] which report higher cumulative incidence of influenza across a season based on serology than RT-PCR and is due to a number of factors: high specificity of RT-PCR, comparatively lower specificity of serology, the greater ability of serology to identify asymptomatic and subclinical infections, and underreporting of symptoms by participants. It is possible for the number of triggered RT-PCR tests required to exceed the number budgeted if more ARI or FARI cases are reported than expected. When this occurred in our simulations, we simulated the cessation of collection or analysis of specimens after the allotted field budget was exhausted. However, if an investigator were able to procure additional funds to collect and analyze the additional specimens required by circumstance this would further increase the power of a design relying on RT-PCR.

These results illustrate that careful planning is necessary when considering the design of cohort studies for influenza. We have purposely intended the present analysis to focus on the design of NPI studies. Our results might be applicable to studies of either vaccine or antiviral prophylaxis with some caveats. In vaccine trials, receipt of the vaccine usually results in higher initial antibody levels following vaccination, making interpretation of paired serology difficult [30], [44]. However our observation that routine collection of respiratory specimens was more efficient than relying on illness trigger may still hold. Regarding antiviral prophylaxis, those receiving treatment could potentially have similar rates of infection as controls but have lower levels and duration of viral shedding and reduced severity of symptoms thus reducing the sensitivity of RT-PCR and clinical definitions [45].

While our results may serve as a broad guideline for investigators planning a cohort study, some limitations exist which may limit their use in practice. First, our models are somewhat sensitive to estimates of serology and RT-PCR costs and their sensitivity and specificity. It is likely that these estimates, especially of costs, would vary geographically. Therefore, we have presented a range of sensitivity analyses varying important model parameters. Second, it is important to note that our results apply only to naturally acquired influenza as opposed to volunteer challenge studies where participants are experimentally exposed to influenza virus [31], [46]. Challenge studies could be more resource efficient than cohort studies in assessing the potential benefits of interventions at preventing infection, although the results may not be generalizable to the use or effectiveness of interventions in natural settings [47]. Third, our results are also specifically based on viral shedding data of influenza A. Influenza B has slightly different epidemiologic characteristics including possibly a longer duration of infectiousness [31], [32]. Finally, we did not consider cluster studies such as school or household-based studies, where optimal design may differ due to correlation in the risk of infection and potential differences in logistics and resources required for fieldwork.

While our study identified RT-PCR as an optimal design by comparing statistical power among different designs as a function of budget, it should be noted that other laboratory testing methods can still be optimal for other clinical or public health objectives. When influenza diagnosis is followed by treatment or public health intervention of potential contacts, testing methods are subject to other considerations. In such an instance, the objectives may be to improve clinical outcome by early treatment or to prevent secondary transmission and thus speed of diagnosis is of critical importance. While our results may not be applicable to this type of study, similar simulation approaches could be used to assess optimal design for other specific objectives under consideration.

In conclusion, large sample sizes are often needed in influenza intervention studies because of the low incidence of influenza and the moderate effects of many interventions. Our results show that a design using biweekly RT-PCR plus ARI trigger, a cumulative incidence of influenza infection of 15% and moderate intervention efficacy of 30%, approximately 1,400 participants (700 to 1800 depending on cumulative incidence of influenza infection) per arm would need to be recruited to achieve 80% power. Further research should continue to improve the accuracy of estimates of the sensitivity and specificity of laboratory methods which would help to improve study power, particularly when comparing serologic and virologic approaches to ascertainment of influenza infections. New laboratory methods may emerge which the accuracy or cost of current techniques and thus would allow for more efficient designs.

## Supporting Information

Text S1 Supplemental Appendix with additional technical details.
(DOC)

Figure S1 **Power of competing influenza diagnostic methods for Scenarios I–III.** Sensitivity analysis (**a**) is when the control arm cumulative incidence is reduced to 0.1 and sensitivity analysis (**b**) is when the control arm cumulative incidence is reduced to 0.3.
(TIF)

Figure S2 **Power of competing influenza diagnostic methods for Scenarios I–III.** Sensitivity analysis (**a**) is when the NPI intervention has no effect on non-influenza ARI and FARI rate but we plan for a 15% reduction and sensitivity analysis (**b**) is when the NPI intervention reduces the non-influenza ARI and FARI rate by 30% but we plan for a 15% reduction.
(TIF)

Figure S3 **Power of competing influenza diagnostic methods for Scenarios I–III.** Sensitivity analysis (**a**) is when the cost of RT-PCR testing is small (US$35) and sensitivity analysis (**b**) when the cost is large (US$130).
(TIF)

Figure S4 **Power of competing influenza diagnostic methods for Scenarios I–III.** Sensitivity analysis (**a**) is when the cost of serology is small (US$130) and sensitivity analysis (**b**) is when the cost of serology is large (US$195).
(TIF)

Figure S5 **Power of competing influenza diagnostic methods for Scenarios I–III.** Sensitivity analysis (**a**) is when the sensitivity of RT-PCR is reduced by 20% (by AUC) and sensitivity analysis (**b**) is when the sensitivity of RT-PCR increased by 10% (by AUC).
(TIF)

Figure S6 **Power of competing influenza diagnostic methods for Scenarios I–III.** Sensitivity analysis (**a**) is when the sensitivity and specificity of serology is reduced to 0.76 and 0.80 respectively and sensitivity analysis (**b**) is when the sensitivity and specificity of serology is increased to 0.92 and 0.96 respectively.
(TIF)

Figure S7 **Power of competing influenza diagnostic methods for Scenarios I–III.** Sensitivity analysis is for a six month follow-up rather than two-months.
(TIF)
