# Supplementary material for: Optimal Design of Intervention Studies to Prevent Influenza in Healthy Cohorts
Source: PLoS One. 2012 Apr 13;7(4):e35166. doi: 10.1371/journal.pone.0035166 (PMC3325991; doi:10.1371/journal.pone.0035166)
Supplement: Text S1 — Supplemental Appendix with additional technical details. (DOC) [file pone.0035166.s001.doc]

**Supplemental Appendix**

**to**

**Optimal design of influenza intervention studies in healthy cohorts**

Parameter values used in the model are given in Table 1. We examined the following seven approaches to identification of influenza infections in the comparative study:

1. Collection and testing by RT-PCR of respiratory specimens from participants reporting FARI.
2. Collection and testing by RT-PCR of respiratory specimens from participants reporting ARI.
3. Collection and testing by RT-PCR of respiratory specimens from all participants at biweekly intervals regardless of illness, as well as from any participants reporting ARI.
4. Collection of paired serum from all participants plus collection and testing by RT-PCR of respiratory specimens from participants reporting FARI.
5. Collection of paired serum from all participants plus collection and testing by RT-PCR of respiratory specimens from participants reporting ARI.
6. Collection of paired serum from all participants plus collection and testing by RT-PCR of respiratory specimens collected from all participants at biweekly intervals regardless of illness, as well as from any participants reporting ARI.
7. Collection of paired serum from all participants.

Simulation algorithm

*Calculation of the sample size:*

The total number of participants enrolled, 2*n*, was calculated for a wide range of fixed field work budgets ranging from US$50,000 to US$5,000,000. Each design utilized equal size, *n*, for both the intervention and control arm. For each study budget and detection method, we calculated the number of subjects which could be recruited. For designs using paired sera, this was straightforward as study cost for each participant was fixed. The sample size was calculated by *n* = total budget/(2*cost per subject).

For designs relying on ARI or FARI trigger, cost per subject could not be fixed before the start of the study because the number of triggered specimen collection is stochastic. When triggered specimen collection was required we calculated the expected number of specimens required per participant. For participants in the control arm this was calculated as the control non-influenza ARI or FARI rate, plus the control cumulative incidence times ARI or FARI sensitivity times the reporting rate (relative to case-ascertained studies). For participants in the intervention arm this was calculated as the control non-influenza ARI or FARI rate times one minus reduction in rate associated with the intervention, plus the control cumulative incidence times one minus the treatment efficacy times ARI or FARI sensitivity times the reporting rate (relative to case-ascertained studies). The expected cost per subject was calculated as the cost of enrollment plus the cost of specimens collected from ARI or FARI times the expected number of specimens triggered. The sample size was then calculated by *n* = total budget/(expected cost per intervention subject + expected cost per control subject).

*Calculation of power*:

We adopt the following procedures to simulation infections within that study population.

1. True attack proportion for the control arm was 15%. Infections were simulated as a stochastic realization from a Bernoulli distribution with event probability 0.15.
2. Treatment efficacy was 30% so the true attack proportion was 10.5% in the intervention arm. Infections were simulated as a stochastic realization from a Bernoulli distribution with event probability 0.105.
3. We assumed that in the control arm participants presented with non-influenza ARI and FARI at rates 0.4 and 0.1 respectively. The number of ARI and FARI for each participant in the control arm was simulated as a stochastic realization from a Poisson distribution with rate parameters 0.4 and 0.1 respectively.
4. We assume the intervention as 15% efficacy against non-influence ARI and FARI. The number of ARI and FARI for each participant in the intervention arm was simulated as a stochastic realization from a Poisson distribution with rate parameters 0.34 and 0.085 respectively.
5. Sixty-eight percent and 40% of true infections were assumed to result in ARI and FARI respectively. Of those we assume 70% percent were reported. Therefore for each true infection the presence of reported ARI and FARI was simulated as a stochastic realization from a Bernoulli distribution with event probabilities 0.476 and 0.28 respectively.
6. For each recorded FARI and ARI episode a date for the episode was generated by a stochastic realization from a Uniform distribution.
7. We assume that the collection of specimens was continued until the study budget was exhausted. It was possible the number of ARI or FARI episodes triggering specimen collection for RT-PCR exceeded the allotted study budget. When this happened, specimens were collected as triggered by ARI or FARI chronologically until the study budget was exhausted.
8. For each specimen collected for RT-PCR analysis that did not arise from a true ARI or FARI episode and for each paired sera collected from a participant who was not infected during the season we simulated the occurrence of a false positive by a stochastic realization from a Bernoulli distribution with event probabilities one minus the specificity (0.01 and 0.12 respectively).
9. For each specimen collected for RT-PCR analysis that arose from an influence ARI or FARI episode and for each paired sera collected from a participant who was infected during the season we simulated the a positive test result by a stochastic realization from a Bernoulli distribution with event probabilities equal to the sensitivity (varies depending on timing but between 0.863 and 0.806 if collection occurs within 3 days of symptom onset and 0.84 respectively).
10. For biweekly testing, we assume that 0.10 percent of specimens are not collected due to refusal from participants. The event of a refusal was simulated using a stochastic realization from a Bernoulli distribution with event probabilities 0.10.
11. For biweekly testing, the probability of detection of a previously unreported case was dependent on the time since acquiring influenza infection and testing (see Figure 1(b)[1]).
12. Using the number of both true and false positives, a chi-squared test was performed to test for group differences. The null hypothesis was that the probability of infections was equal in the intervention and control arms. If a p-value of less than or equal to 0.05 was observed, the null hypothesis was rejected.

This procedure was repeated 2500 times. This procedure was also used for each sensitivity analysis varying specified parameters. The statistical power was calculated as the number of times the null hypothesis was rejected at a significance level of 0.05, divided by 2500. All simulations were conducted in R version 2.10.1 (R Development Core Team, Vienna, Austria).

1. Klick B, Leung GM, Cowling BJ (2011) Optimal design of studies of influenza transmission in households. I: Case-ascertained studies. Epidemiol Infect: 1-9.
