# Supplementary figures and images for: Optimal Design of Intervention Studies to Prevent Influenza in Healthy Cohorts
Source: PLoS One. 2012 Apr 13;7(4):e35166. doi: 10.1371/journal.pone.0035166 (PMC3325991; doi:10.1371/journal.pone.0035166)

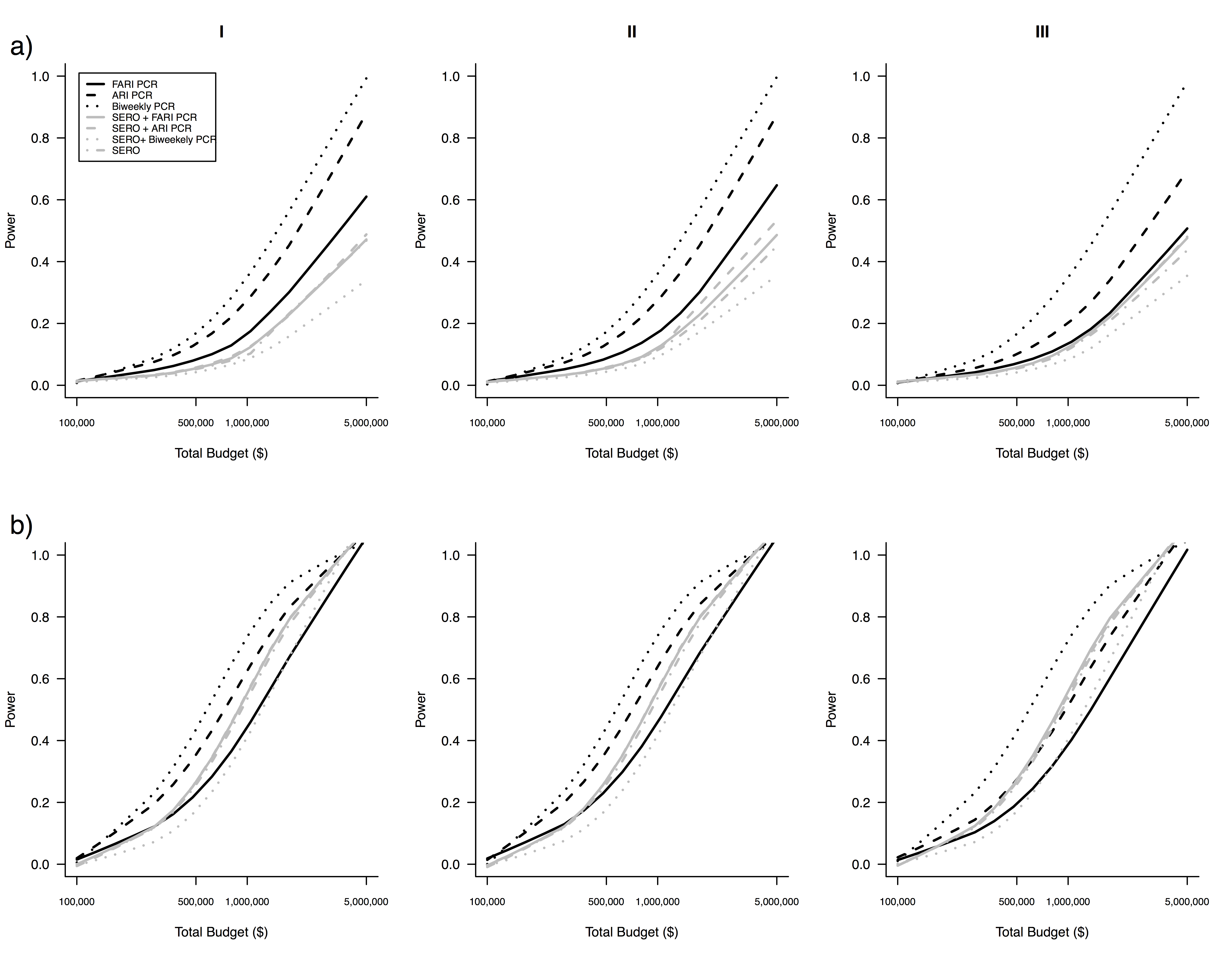

Supplement: Figure S1 — Power of competing influenza diagnostic methods for Scenarios I–III. Sensitivity analysis (a) is when the control arm cumulative incidence is reduced to 0.1 and sensitivity analysis (b) is when the control arm cumulative incidence is reduced to 0.3. (TIF) [file pone.0035166.s002.tif]

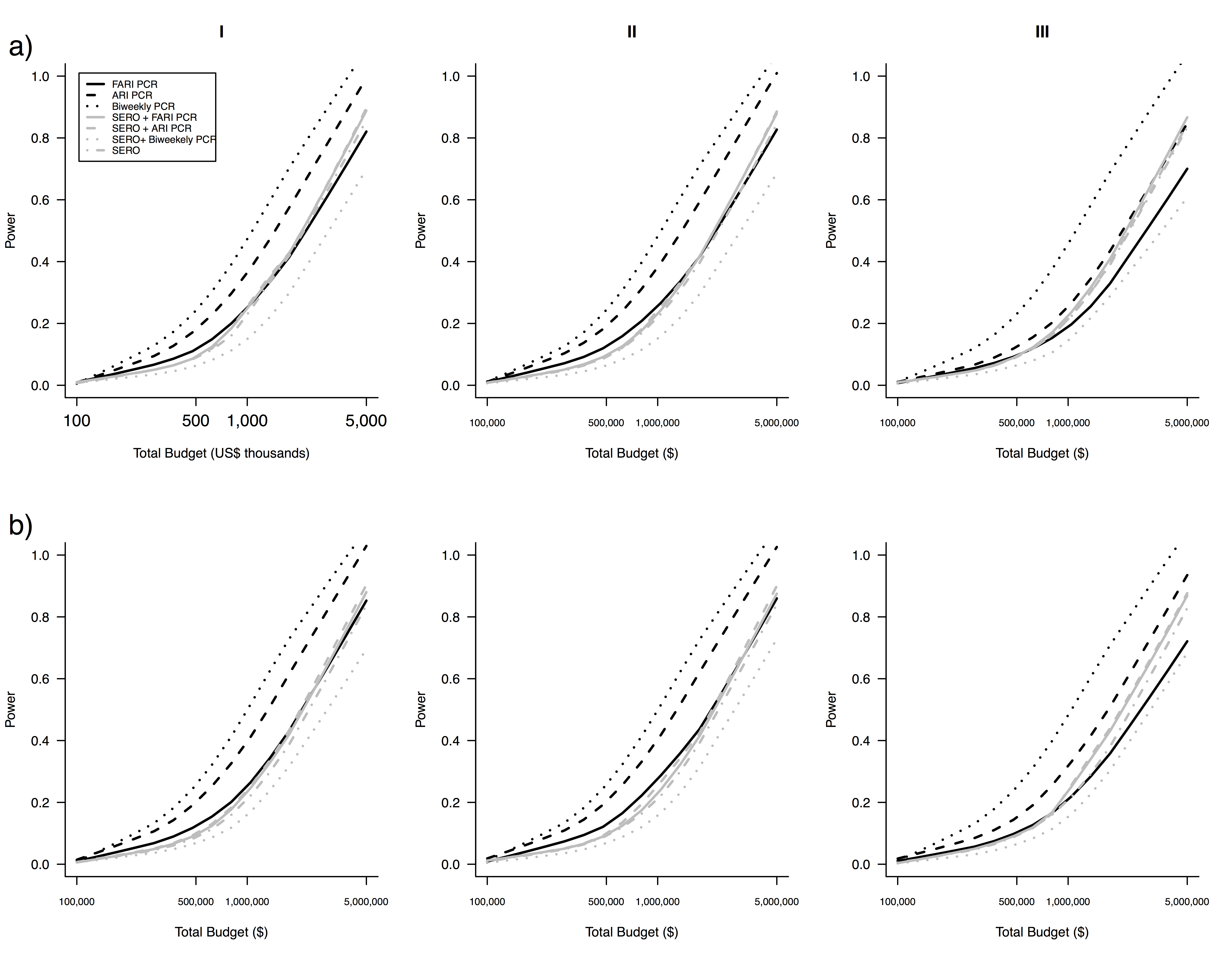

Supplement: Figure S2 — Power of competing influenza diagnostic methods for Scenarios I–III. Sensitivity analysis (a) is when the NPI intervention has no effect on non-influenza ARI and FARI rate but we plan for a 15% reduction and sensitivity analysis (b) is when the NPI intervention reduces the non-influenza ARI and FARI rate by 30% but we plan for a 15% reduction. (TIF) [file pone.0035166.s003.tif]

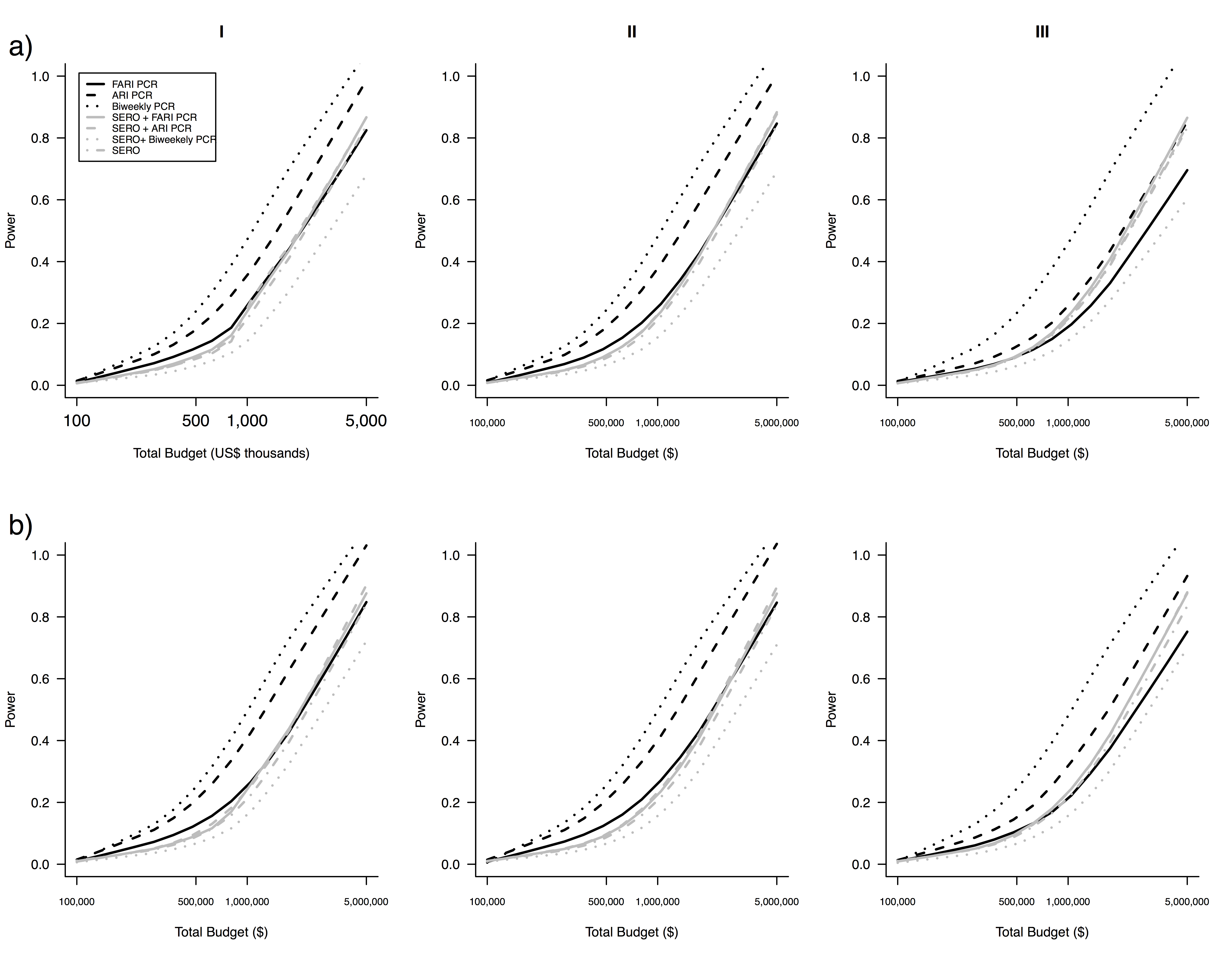

Supplement: Figure S3 — Power of competing influenza diagnostic methods for Scenarios I–III. Sensitivity analysis (a) is when the cost of RT-PCR testing is small (US$35) and sensitivity analysis (b) when the cost is large (US$130). (TIF) [file pone.0035166.s004.tif]

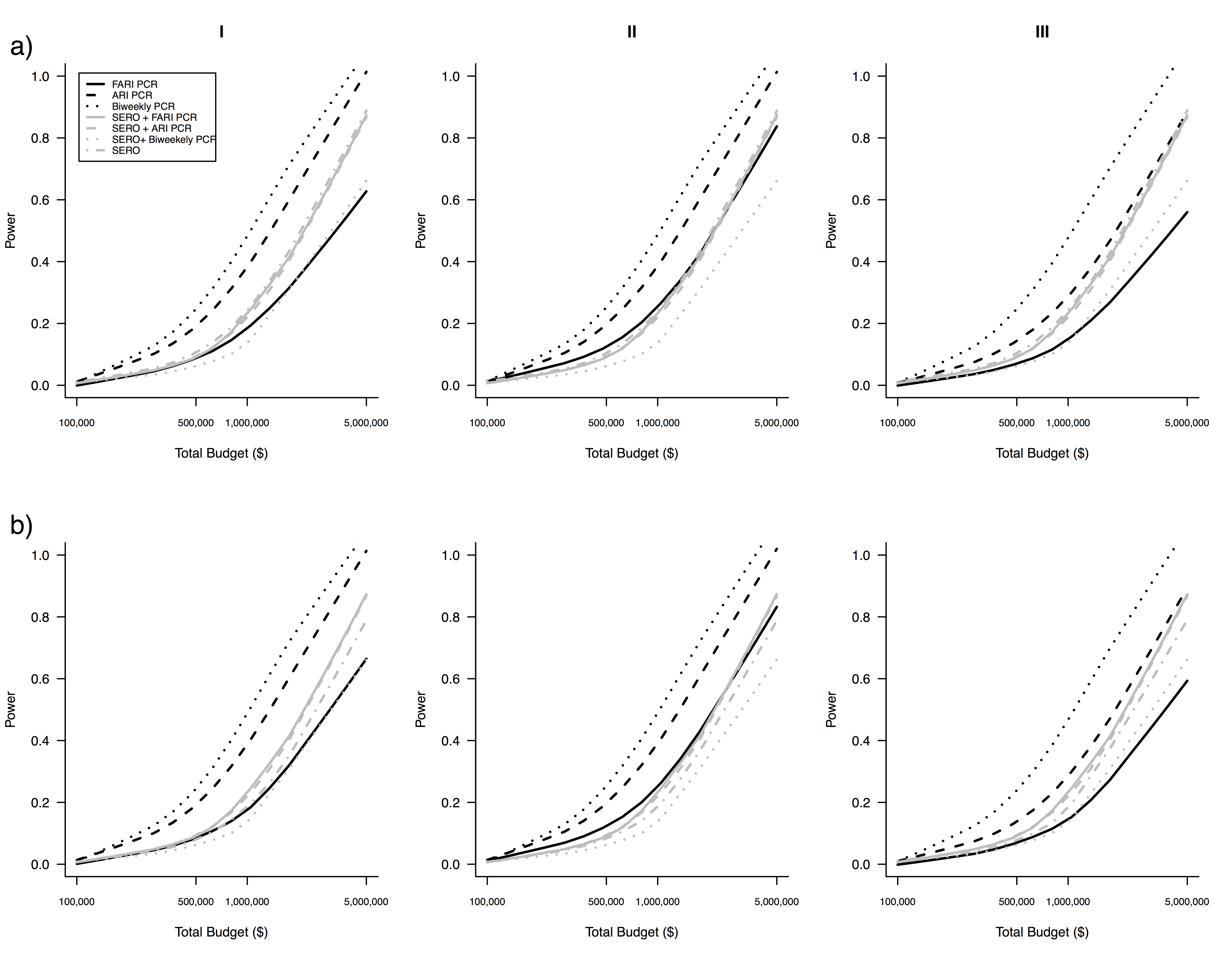

Supplement: Figure S4 — Power of competing influenza diagnostic methods for Scenarios I–III. Sensitivity analysis (a) is when the cost of serology is small (US$130) and sensitivity analysis (b) is when the cost of serology is large (US$195). (TIF) [file pone.0035166.s005.tif]

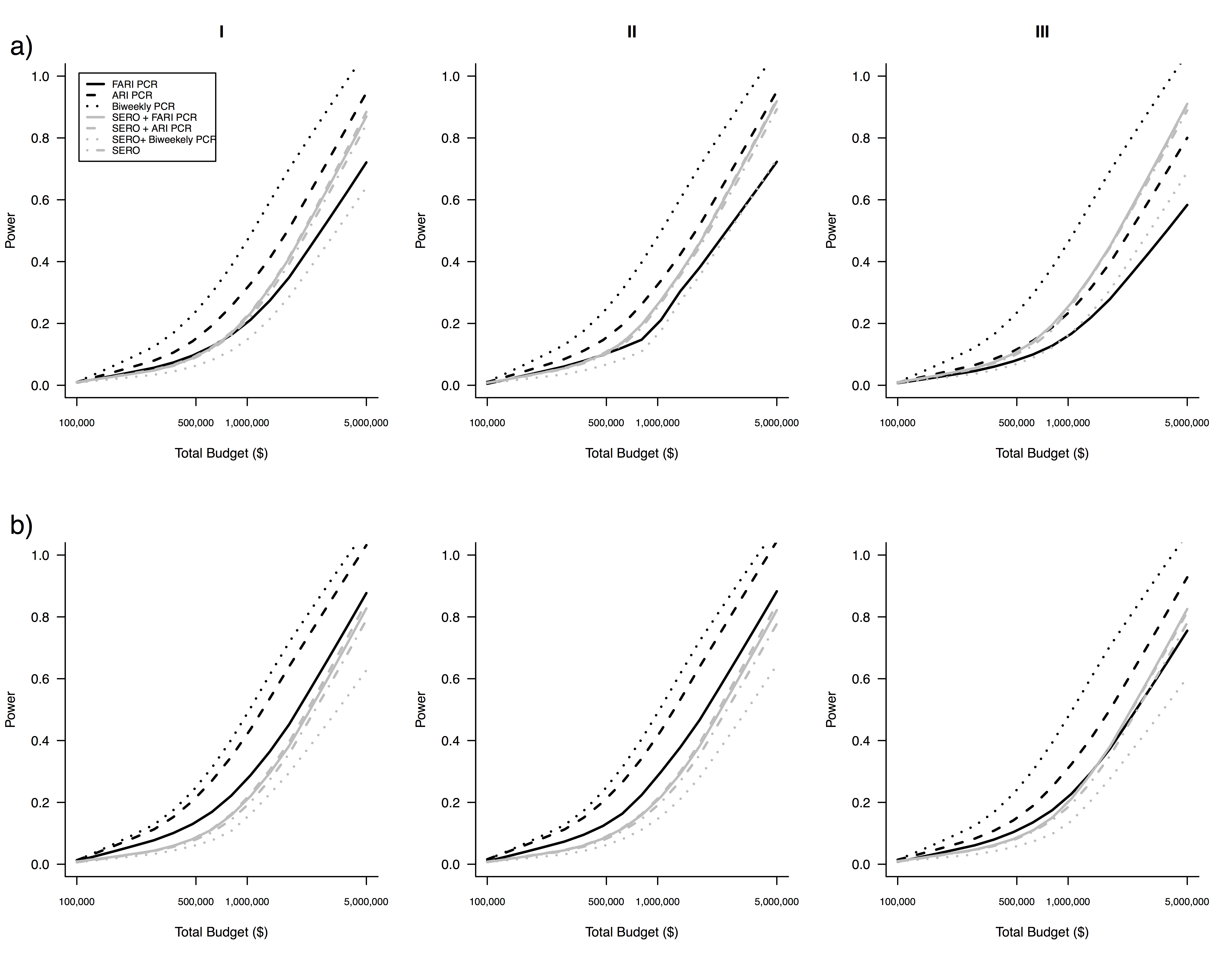

Supplement: Figure S5 — Power of competing influenza diagnostic methods for Scenarios I–III. Sensitivity analysis (a) is when the sensitivity of RT-PCR is reduced by 20% (by AUC) and sensitivity analysis (b) is when the sensitivity of RT-PCR increased by 10% (by AUC). (TIF) [file pone.0035166.s006.tif]

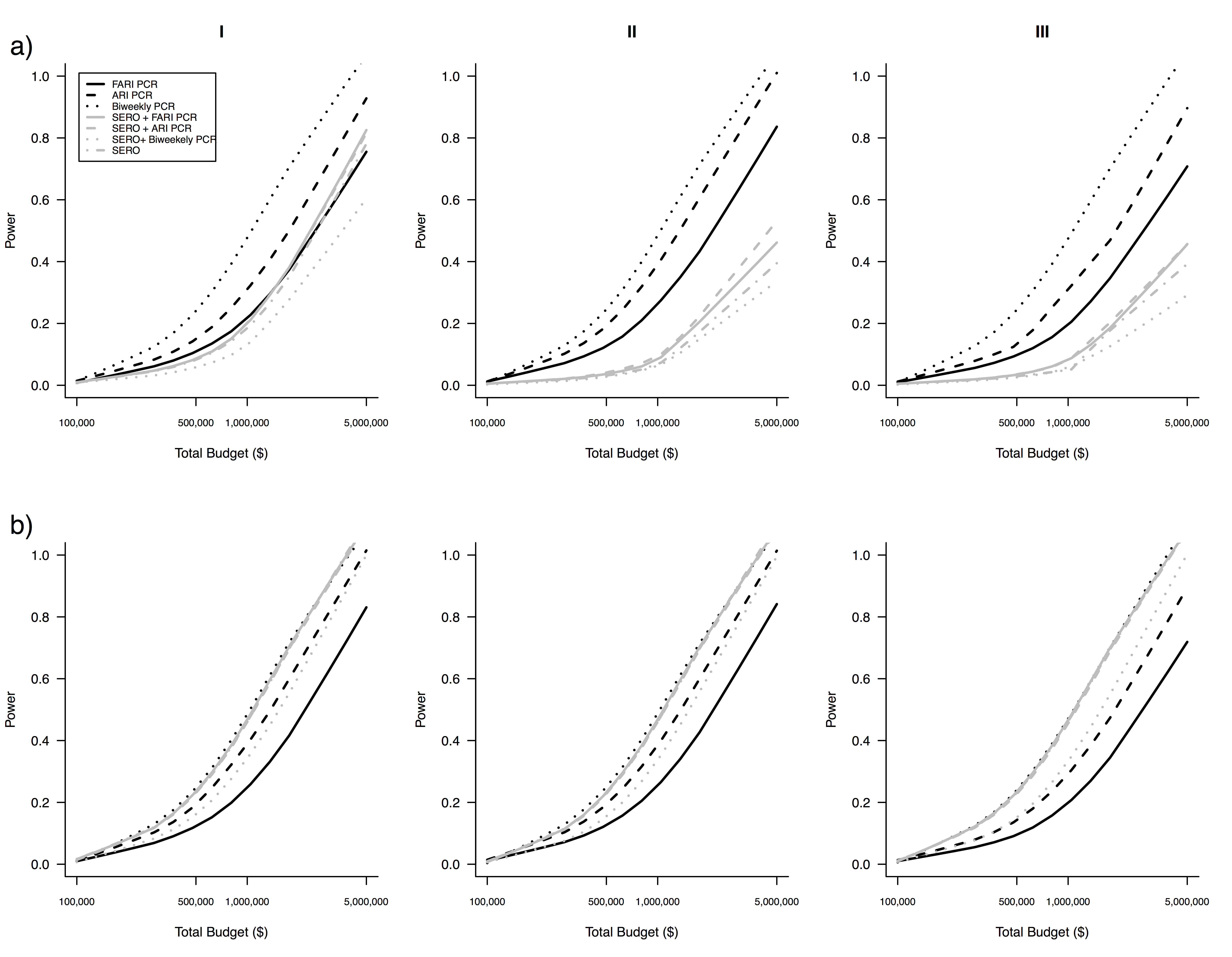

Supplement: Figure S6 — Power of competing influenza diagnostic methods for Scenarios I–III. Sensitivity analysis (a) is when the sensitivity and specificity of serology is reduced to 0.76 and 0.80 respectively and sensitivity analysis (b) is when the sensitivity and specificity of serology is increased to 0.92 and 0.96 respectively. (TIF) [file pone.0035166.s007.tif]

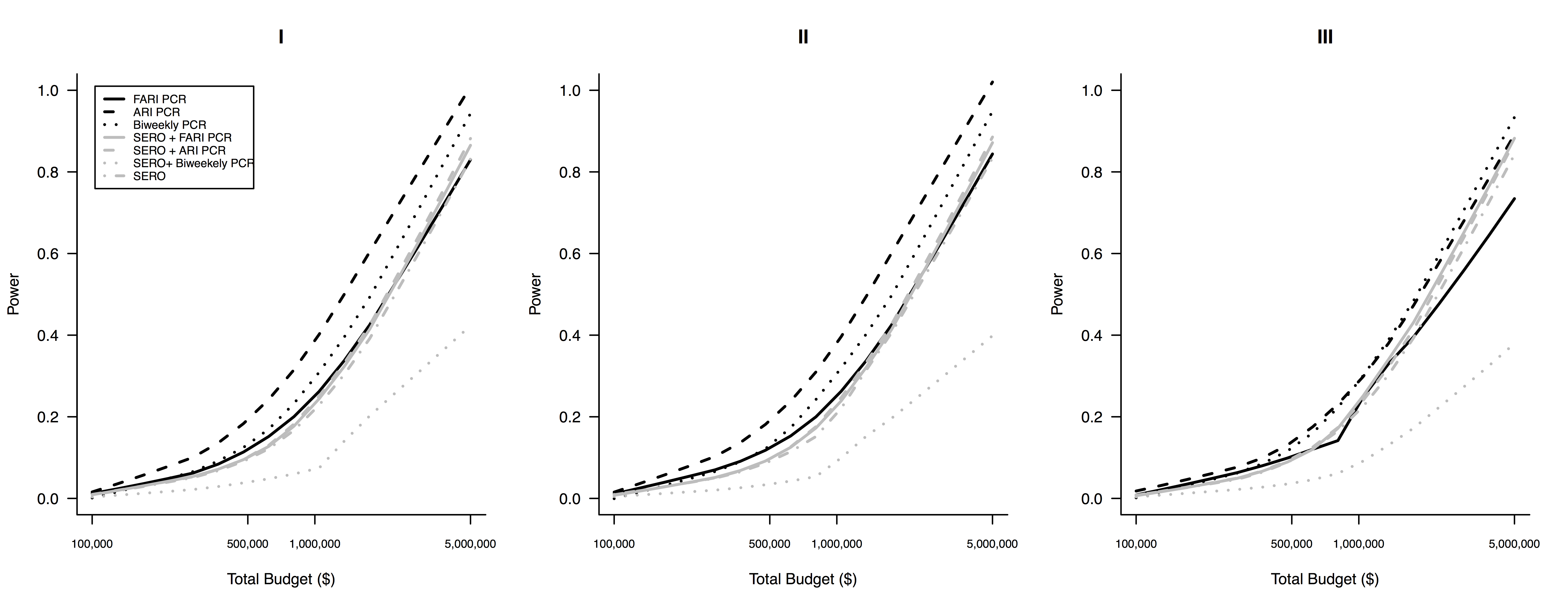

Supplement: Figure S7 — Power of competing influenza diagnostic methods for Scenarios I–III. Sensitivity analysis is for a six month follow-up rather than two-months. (TIF) [file pone.0035166.s008.tif]
